# Tumor-specific major histocompatibility-II expression predicts pathological complete response to atezolizumab combined to chemotherapy in triple-negative breast cancer

**DOI:** 10.1038/s41523-025-00828-6

**Published:** 2025-09-29

**Authors:** Justin M. Balko, Luca Licata, Xiao Qian Wang, Matteo Dugo, Chiun-Sheng Huang, Daniel Egle, Begoña Bermejo, Claudio Zamagni, Marc Thill, Antonio Anton, Stefania Russo, Elena Sevillano, Eva Maria Ciruelos, Richard Greil, Vladimir Semiglazov, Marco Colleoni, Catherine M. Kelly, Gabriella Mariani, Lucia Del Mastro, Stefania Zambelli, Giulia Viale, Maurizio Callari, Giuseppe Viale, Lajos Pusztai, Luca Gianni, H. Raza Ali, Giampaolo Bianchini

**Affiliations:** 1 Department of Medicine, Vanderbilt University Medical Center, Nashville, TN USA; 2 Department of Medical Oncology, IRCCS San Raffaele Hospital, Milan, Italy; 3 CRUK Cambridge Institute, University of Cambridge, Cambridge, UK; 4 National Taiwan University Hospital and College of medicine, National Taiwan University, Taipei, Taiwan; 5 Department of Gynecology, Medical University Innsbruck, Innsbruck, Austria; 6 Medical Oncology, Hospital Clínico Universitario de Valencia, Biomedical Research Institute INCLIVA, Valencia, Spain; 7 Medicine Department, Universidad de Valencia, Valencia, Spain; 8 Oncology Biomedical Research National Network (CIBERONC-ISCIII), Madrid, Spain; 9 IRCCS Azienda Ospedaliero-universitaria di Bologna, Municipality, Italy; 10 Department of Gynecology and Gynecological Oncology Agaplesion Markus Krankenhaus, Frankfurt, Germany; 11 Hospital Universitario Miguel Servet, Instituto de Investigación Sanitaria Aragón (IISA), Universidad de Zaragoza, Zaragoza, Spain; 12 Department of Oncology, Santa Maria della Misericordia University Hospital, Azienda Sanitaria Universitaria Friuli Centrale (ASUFC), Udine, Italy; 13 HM CIOCC MADRID (Centro Integral Oncológico Clara Campal), Laboratorio de Innovación en Oncología, Instituto de Investigación Sanitaria HM Hospitales, Madrid, Spain; 14 Hospital Universitario 12 de Octubre, Madrid, Spain; 15 Paracelsus Medical University Salzburg, Salzburg Cancer Research Institute-Center for Clinical Cancer and Immunology Trials, Cancer Cluster Salzburg, Salzburg, Austria; 16 N. N. Petrov Research Institute of Oncology, St. Petersburg, Russian Federation; 17 Division of Medical Senology, IEO European Institute of Oncology, IRCCS, Milan, Italy; 18 Department of Medical Oncology, Mater Private Hospital, Dublin, Ireland; 19 Fondazione IRCCS Istituto Nazionale dei Tumori, Milan, Italy; 20 Clinical Oncology Unit; IRCCS Ospedale Policlinico San Martino, Genova, Italy; 21 Department of Internal Medicine and Medical Specialties; Università di Genova, Genova, Italy; 22 Fondazione Michelangelo, Milan, Italy; 23 Department of Pathology & Laboratory Medicine, IEO European Institute of Oncology, IRCCS, Milan, Italy; 24 Yale Cancer Center, Yale School of Medicine, New Haven, CT USA; 25 International Breast Cancer Research Committee—Fondazione Michelangelo, Milan, Italy; 26 School of Medicine and Surgery, Università Vita-Salute San Raffaele, Milan, Italy

**Keywords:** Biomarkers, Cancer, Immunology, Oncology

## Abstract

Adding immune checkpoint inhibitors to neoadjuvant chemotherapy improves outcomes in early-stage triple-negative breast cancer (TNBC), but a fraction of patients derive benefit. Tumor-specific MHC-II (tsMHC-II) expression has been shown to be a predictive biomarker of pathological complete response (pCR) to neoadjuvant chemo-immunotherapy in early-stage TNBC. We performed biomarker analysis of the phase III NeoTRIP trial where patients were randomized to neoadjuvant carboplatin and nab-paclitaxel±atezolizumab. Imaging mass cytometry was used to assess tsMHC-II expression in tumor samples. TsMHC-II positivity was predefined as ≥5% of tumor cells expressing MHC-II, and at an 80th percentile exploratory cutoff. TsMHC-II positivity was associated with a higher pCR rate in the atezolizumab arm (OR:2.58; *P* = 0.016), but not in the chemotherapy-only arm (OR:1.37; *P* = 0.34) and these results were stronger using the exploratory cutoff. TsMHC-II expression is associated with improved response to neoadjuvant chemo-immunotherapy in early TNBC and could represent a clinically useful predictive biomarker for treatment personalization.

## Introduction

The anti-PD-1 monoclonal antibody pembrolizumab, an immune checkpoint inhibitor (ICI), is approved for treatment of high-risk early triple-negative breast cancer (TNBC)^1,2^ in combination with chemotherapy and broadly recognized as standard of care. However, less than 10% of the patients derive individual benefit from the addition of pembrolizumab to neoadjuvant chemotherapy (NAC). ICIs can cause chronic immune related adverse events, including severe or even fatal toxicities such as myocarditis, pneumonitis and encephalitis^3^. Therefore, biomarkers that can identify patients that benefit from the addition of ICIs are needed^4^.

Despite various promising biomarkers, none have been validated to demonstrate clinical utility in early stage TNBC^5^^,^. Tumor-specific major histocompatibility complex-II (tsMHC-II) has been identified as a potential biomarker for PD-1/PD-L1 targeted immunotherapy in other cancer types including melanoma^6,7^, Hodgkin lymphoma^8^ and bladder cancer^9^. MHC-II has multiple isotypes: HLA-DR, HLA-DP, and HLA-DQ, each with alpha and beta subunits that form heterodimers. MHC-II is constitutively expressed on the surface of antigen presenting cells and loaded with exogenously derived peptides to be presented to CD4 + T-cells to regulate immune responses. However, MHC-II can also be expressed on normal and cancer cells including breast epithelial cells and tumors^10^, although its function in this setting is not well-established.

TsMHC-II has been independently identified as a predictor of molecular response to single-agent anti-PD-1 in a small window trial in the early breast cancer setting using a biomarker screening approach^11^. TsMHC-II expression was assessed retrospectively on samples from patients with high-risk early-stage HR + HER2- and TNBC that participated in one of two Phase II clinical trials evaluating the addition of pembrolizumab^12^ or durvalumab^13^ to NAC. Using a pre-defined cutoff of ≥5% of tumor cells by immunohistochemistry, or a harmonized equivalent cutoff by an orthogonal method (reverse phase protein array/RPPA), tsMHC-II was confirmed to be predictive of pCR and event-free survival (EFS) to PD-1/PD-L1 inhibitors in addition to NAC but not to NAC alone^14^. Here, using the pre-defined 5% tumor positivity cutoff we tested the predictive value of tsMHC-II to predict pCR in the Phase III NeoTRIPaPDL1 trial^15^.

## Results

### Tumor-specific MHC-II expression predicts ICI benefit

A total of 220 FFPE tissues at baseline were available for the present analysis: 109 in the chemotherapy arm and 111 in the chemotherapy plus immunotherapy arm. Using the predefined cutoff of 5% tumor-cell positivity, we found that 41% of all breast cancers assessed expressed ≥5% MHC-II, with no significant differences between treatment arms (42.2% in the chemotherapy alone group and 40.5% in the chemotherapy plus immunotherapy group, respectively; *p* = 0.68) (Table 1).

**Table 1:** Distribution of MHC-II-positive tumors according to 5% and 80% cutoffs

	MHC-II groups			
Treatment arm - no. (%)	<5%	>5%	<80th percentile	>80th percentile
**CT**	63 (57.8)	46 (42.2)	90 (82.6)	19 (17.4)
**CT/A**	66 (59.5)	45 (40.5)	86 (77.5)	25 (22.5)

**Fig. 1:**
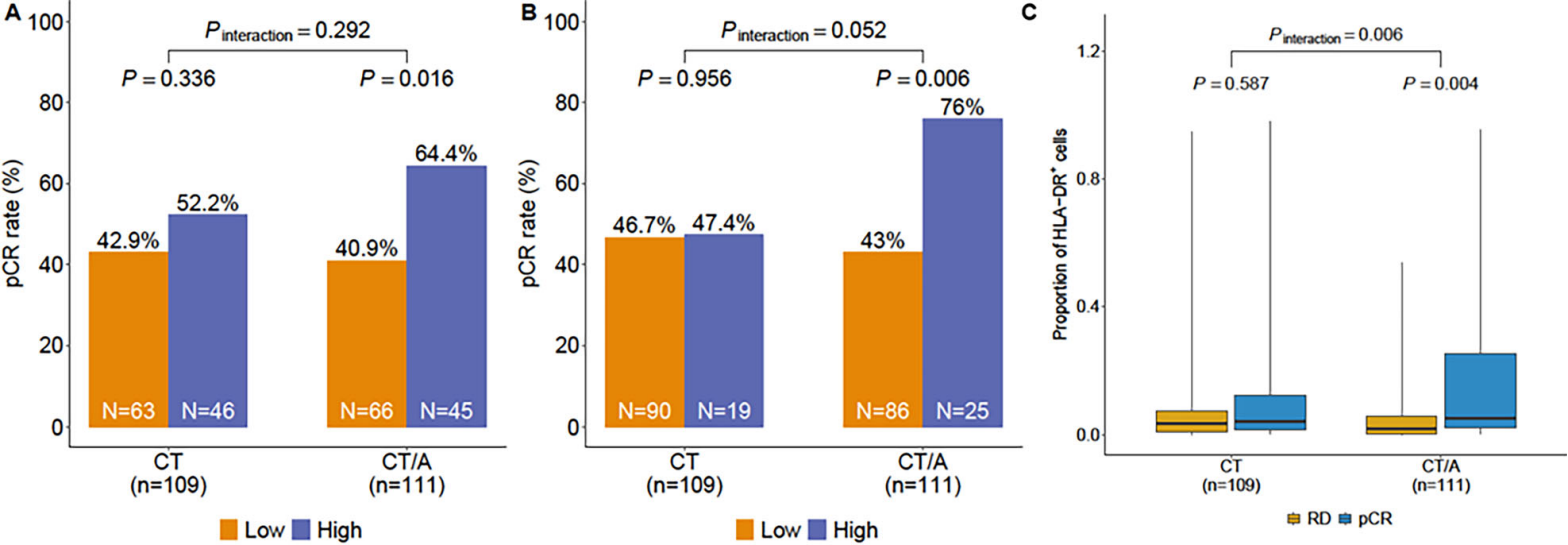
Association of MHC-II with pathological response to chemoimmunotherapy. Association of tumor-specific MHC-II expression with outcome to atezolizumab and chemotherapy or chemotherapy alone using the cutoffs of 5% (**A**) and the 80th percentile (**B**) tumor-cell positivity. **C** tsMHC-II measured continuously in treatment/response arms.

The application of this cutoff point demonstrated predictive capacity for pCR in the immunotherapy-treated patients (one-tailed chi-square test, *p* = 0.016, OR: 2.58) but not in the chemotherapy alone arm (*p* = 0.336, OR: 1.45). The test for interaction between MHC-II status and treatment arm on pCR was not significant (*p* = 0.292) (Fig. 1A).

Since the predefined 5% cutoff identified significantly more positive patients using IMC than expected with immunofluorescence or the reverse phase protein array quantification equivalent (15–20%)^14^, we conducted an exploratory analysis to test the association between tsMHC-II status and pCR rates for each treatment, using the cutoff of the 80th percentile of tumor-cell positivity, to mirror a similar split proportion. With this cutoff point, we found that 17.4% and 22.5% of breast cancers in the chemotherapy alone and chemotherapy plus immunotherapy arms, respectively, were tsMHC-II+ (*p* = 0.35) (Table 1). TsMHC-II status continued to demonstrate its predictive capacity for pCR in the immunotherapy-treated patients (*p* = 0.006, OR: 4.19), but not in the chemotherapy alone arm (*p* = 0.956, OR: 1.03). Moreover, when testing the interaction between MHC-II status using this cutoff and treatment arm on pCR, we observed a trend toward significance (*p* = 0.052) (Fig. 1B). Measuring tsMHC-II continuously demonstrated a statistically significant interaction between treatment arms and outcome groups (*p* = 0.006) (Fig. 1C). Receiver-operator characteristic analysis also demonstrated an improved area-under-the-curve across cut points in the chemo-immunotherapy arm over chemotherapy alone (Supplementary Fig. 1).

## Discussion

Here, we have demonstrated continued support for the predictive value of tsMHC-II to identify an early-stage TNBC patient population who seems to benefit the most from immunotherapy, in the context of a randomized, controlled Phase III trial. While the antibody used for MHC-II detection in situ is consistent with prior studies, the IMC method utilized herein is more sensitive that traditional IHC, immunofluorescence, and RPPA methodologies used in previous analyses^6,14^. Thus, a modified cut-off identifying a similar percentage of patients as ‘positive’ for the biomarker (20%) by IHC was also tested. Both analyses demonstrated the clinical validity of tsMHC-II as predictive biomarker of immunotherapy benefit in this setting. The planned IMC approach for assessing multiple parameters, including MHC-II, preceded the reporting of tsMHC-II as an immunotherapy biomarker in breast cancer, and remaining tissue resources were too limited to reasonably conduct direct IHC on the cohort, and thus this analysis can be considered an “analysis of convenience”.

The results from this study, and prior validations of tsMHC-II, suggest its utility as a specific predictor of immunotherapy benefit in early-stage TNBC. This is in contrast to stromal TILs and PD-L1, which have instead shown clear prognostic value or predictive value to chemotherapy^16–23^, and have shown lack of utility in selecting immunotherapy-specific benefit in randomized controlled trials^1^^,^^24,25^. Studies suggesting otherwise often lack a chemotherapy-backbone control, making such an assessment difficult to parse from prognostic or broadly predictive effects.

As additional limitations, atezolizumab and the chemotherapy regimen used in the NeoTRIP trial are different from the current approved regimen, and EFS is not available yet for translational studies. However, the consistency of these results support this as a promising biomarker with potential clinical utility to be demonstrated in correlative analyses of ongoing clinical trials. TsMHC-II is currently under evaluation as a planned integrated biomarker on multiple prospective phase III clinical trials evaluating the addition of pembrolizumab to chemotherapy in the adjuvant setting for patients with TNBC (S1418; NCT02954874) and in the neoadjuvant settings (S2206; NCT06058377) for patients with high-risk ER+ early breast cancer. We are continuing to work on harmonizing tsMHC-II assays to define the most practical approach that can be prospectively validated in a pre-planned and defined fashion to demonstrate true clinical utility, with careful consideration of establishing the most widely-available assay that can be performed in the most technically rigorous way.

## Methods

### Study design and prospective tissue collection

Breast tumor samples were obtained from patients enrolled in the multi-center, randomized, open-label, phase III NeoTRIP trial^15^. In NeoTRIP, 280 patients with early high-risk TNBC were randomized to receive eight cycles of neoadjuvant carboplatin and nab-paclitaxel on days 1 and 8 every 3 weeks, with or without atezolizumab on day 1. Following neoadjuvant therapy, patients underwent surgery, to be followed by four cycles of post-surgery anthracyclines at the treating clinician’s discretion. The Per-Protocol Population (*n* = 258) was used for all translational studies. Core biopsies for research were obtained at baseline, after one cycle of therapy, and at surgery. Only baseline biopsies were used for the present analysis. The study was undertaken in accordance with Good Clinical Practice guidelines and the Declaration of Helsinki. All patients provided written informed consent. Approvals for the study protocol (and any modifications thereof) were obtained from independent ethics committees at each participating institution and relevant competent authorities. The following institutions and ethics committees participated: Brustgesundheitzentrum Tirol, Univ. Frauenklinik Innsbruck, Innsbruck, Austria 6020; Universitätsklinik für Innere Medizin III, mit Hämatologie, internistischer Onkologie, Hämostaseologie, Infektiologie, Rheumatologie und Onkologisches Zentrum Salzburg, Austria 5020; Klinikum Augsburg International Patient Service Augsburg, Germany, 86156; Frauenarzt-Zentrum-Zehlendorf, Berlin, Germany, 14169;Augusta-Kranken-Anstalt gGmbH Klinik für Hämatologie, Onkologie & Palliativmedizin, Bochum, Germany, 447891;Bethanien-Krankenhaus Onkologisches Zentrum, Frankfurt, Germany, 60389;Markus Krankenhaus Klinik für Gynäkologie und Geburtshilfe, Frankfurt, Germany, 60431;Gynäkologisch-Onkologische Praxis, Hannover, Germany, 30177;NCT Nationales Centrum für Tumorerkrankungen, Heidelberg, Germany, 69120;Uniklinik Köln Klinik und Poliklinic für Frauenheilkunde und Geburtshilfe Brestzentrum, Köln, Germany, 50931;Brustzentrum St. Elisabeth-Krankenhaus, Köln, Germany, 50935;Interdisciplinary Oncology Center (IOZ), Munchen, Germany, 80336;Cork University Hospital, Cork, Ireland;Beaumont Hospital, Dublin, Ireland;Mater Misericordiae University Hospital, Dublin, Ireland;St. James’s Hospital, Dublin, Ireland;University Hospital Waterford, Waterford, Ireland;Policlinico S. Orsola Malpoghi, Bologna, Italy, 40138;Istituto per la Ricerca sul Cancro, Candiolo, Italy, 10060;IST San Martino, Genova, Italy, 16132;Istituto Toscano Tumori Ospedale Misericordia, Grosseto, Italy, 58100;Ospedale San Raffaele, Milano, Italy, 20132;Fondazione IRCCS Istituto nazionale dei Tumori, Milano, Italy, 20133;Istituto Europeo di Oncologia, Milano, Italy, 20141;Ospedale Luigi Sacco, Milano, Italy, 20160;Arcispedale Santa Maria Nuova - A.O. Reggio Emilia, Reggio Emilia, Italy, 42123;Ospedale Santa Maria della Misericordia, Udine, Italy, 33100;Russian Cancer Research Center named after N.N.Blokhin, Moscow, Russian Federation; Petrov Research Institute of Oncology, Department of Breast Cancer, Saint Petersburg, Russian Federation;Road clinical hospital of OJSC “Russian Railways”, Saint Petersburg, Russian Federation;Hospital Duran i Reynal Institut Català d’Oncologia, Hospitalet de Llobregat, Spain, 08908; Hospital Clínico San Carlos, Madrid, Spain, 28040; Hospital Universitario 12 de octubre, Madrid, Spain, 28041; Hospital Universitario HM Sanchinarro, Centro Integral Oncologico Clara Campal (CIOCC), Madrid, Spain, 28050; Hospital Clinico Universitario de Valencia Servicio de Onco-Hematologia, Valencia, Spain, 46010; Hospital Miguel Servet Zaragoza, Spain, 59009; C. Christian Hospital Taiwan, Changhua City, Taiwan; Kaohsiung Medical University Hospital, Kaohsiung, Taiwan; China Medical University Hospital No.2, Taichung City, Taiwan; National Taiwan University Hospital, Taipei, Taiwan;Veteran General Hospital Taipei, Taipei, Taiwan

### Imaging mass cytometry

Imaging mass cytometry (IMC) was used to profile the expression of 43 proteins at subcellular resolution in formalin-fixed paraffin-embedded (FFPE) tumor samples. Detailed protocols of IMC analysis have been reported elsewhere^26^. Briefly, three ROIs measuring 500 × 500 μm2 were identified in core biopsies by a breast pathologist using the Aperio eSlideManager web application (Leica Biosystems).

Cell phenotypes were assigned by semi-supervised clustering. Cells were first classified as epithelial or tumor microenvironment (TME) cells using multiple classification methods. Thresholds were identified for assigning a cell as ‘positive’ for a given marker by inspecting a random selection of at least 50 images for which cells passing a quantile threshold (calculated using all data) were highlighted. This procedure was repeated at differing quantile thresholds until the value that most closely aligned with marker positivity was identified. For this study, MHC-II-positive tumor cells were identified as the cells expressing the HLA-DR antigen, detected by using the anti-mouse monoclonal antibody TAL1B5 (Abcam) at concentration of 0.5 μg/mL. Although TAL1B5 is labeled to recognize only HLA-DR, prior studies by our group have shown extremely high concordance across tumors with pan-MHC-II (HLA-DR/DP/DQ/DX) antibodies^6^, and the TAL1B5 antibody is widely used and provides extremely robust signals at high dilutions. Moreover, MHC-II gene products are widely accepted to be co-regulated by CIITA activation, and therefore are nearly always expressed coordinately with one another, albeit at different levels^27^.

### Statistical analysis

We aimed to evaluate whether the proportion of tumor cells with positive MHC-II status predicts the benefit of adding immunotherapy to NAC. We evaluated pCR rates^15^ as outcome measure, because the EFS results for the NeoTRIP trial have not been published yet. Consistently with previous studies, MHC-II status was initially defined according to a cutoff of 5% tumor cell-positivity^14^. A modified cutoff was also tested to account for the differences in detection method sensitivity (imaging mass cytometry versus IHC) and antibodies. Association between MHC-II status and pCR rates for each treatment arm and test of interaction were assessed using logistic regression. The statistical analyses included herein were not preplanned per study protocol but were planned as exploratory correlative endpoints. Statistical analyses were performed in R version 4.2.1.

## Supplementary information

Supplementary Information

## Data Availability

All imaging mass cytometry and clinical response data can be accessed via a Zenodo data repository (10.5281/zenodo.7990870) for academic non-commercial research.
