# Supplementary material for: Tumor-specific major histocompatibility-II expression predicts pathological complete response to atezolizumab combined to chemotherapy in triple-negative breast cancer
Source: NPJ Breast Cancer. 2025 Sep 29;11:103. doi: 10.1038/s41523-025-00828-6 (PMC12480687; doi:10.1038/s41523-025-00828-6)

**Supplementary Figure 1: ROC curve analysis using a continuous measurement of tumor-specific MHC-II. A) Atezolizumab arm; B) Chemotherapy-alone arm.**


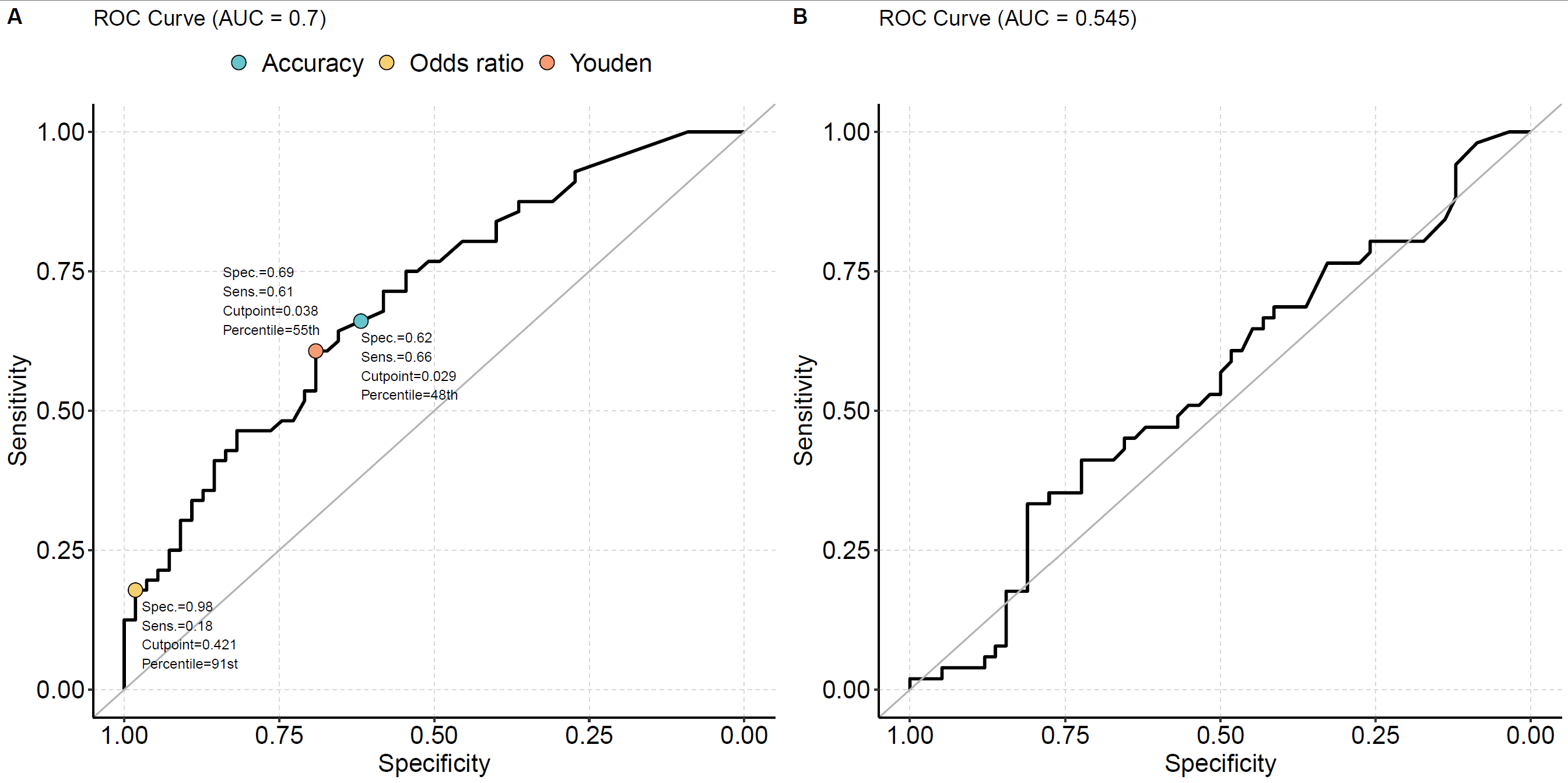

Supplement: Supplementary file 1 — Supplementary Information [file 41523_2025_828_MOESM1_ESM.docx]
